# Co-Producing a Community Follow-Up to SAFER-MH: Strengthening Safety and Continuity of Care After Discharge from Inpatient Mental Health Services

**DOI:** 10.1192/bjo.2026.11139

**Published:** 2026-06-30

**Authors:** Bianca Ungureanu, Natasha Tyler, Maria Panagioti

**Affiliations:** 1 University of Birmingham, Birmingham, United Kingdom; 2 University of Manchester, Manchester, United Kingdom

## Abstract

**Aims:**

Patients discharged from inpatient mental health services can experience multiple adverse outcomes, including a 191-fold higher suicide risk within three months of discharge from inpatient mental health services. The SAFER-MH intervention was co-designed to improve quality and safety in this vulnerable period. Preliminary research suggests it is feasible, acceptable, and offers vital support during inpatient and pre-discharge phases. However, its use is limited to secondary care. This study aimed to strengthen SAFER-MH by co-producing a community follow-up element of the intervention to enhance continuity of care post-discharge.

**Methods:**

A three-phase sequential design was used. Phase 1 involved focus groups with patients, carers, and professionals from primary and community care to explore current practices. Phase 2 used a Nominal Group Technique (NGT) workshop to generate and prioritise feasible solutions. Phase 3 consisted of a co-design workshop with the stakeholder groups described above to collaboratively refine and assess the feasibility of proposed interventions.

**Results:**

Seventeen participants took part across three workshops: Phase 1 included fourindividuals from three abovementioned stakeholder groups; Workshops 2 and 3 added three inpatient professionals and two replacements for those who withdrew. Six themes emerged in Phase 1, including fragmented communication and coordination between services, inadequate discharge preparedness, service limitations and inequalities, patient/carer related barriers, quality and safety concerns and pharmacological care challenges. The NGT workshop generated 33 potential solutions, with 17 receiving votes. Top priorities included a one-stop hub and peer support, assuming no resource or implementation constraints. In Phase 3, participants focused on which elements would be feasible as part of SAFER-MH and the top three interventions included adopting a true multidisciplinary team approach to care, improving medication follow-up, and devising a community peer support element.

**Conclusion:**

This study highlighted shared problems faced by all stakeholder groups post-discharge centring around communication breakdowns, medication challenges, and limited patient involvement. Integrating multidisciplinary teamwork, medication follow-up, and peer support within SAFER-MH is feasible and could enhance continuity and safety during transitions from inpatient to community care.

This study was co-designed and co-produced with patient and public contributors and key stakeholders throughout all stages, resulting in a strengthened SAFER-MH intervention that is now ready for evaluation in a national randomised controlled trial.

